# Characteristics, Opportunities, and Challenges of Osteopathy Based on the Perceptions of Osteopaths in Austria: Qualitative Interview Study

**DOI:** 10.2196/45302

**Published:** 2024-01-17

**Authors:** Jonas Manschel, Jan Porthun, Ulrike Winkler, Jean Marie A T Beuckels, David Martin

**Affiliations:** 1 Institute of Integrative Medicine Department of Medicine, Health Faculty University Witten/Herdecke Herdecke Germany; 2 Norwegian University of Science and Technology, Campus Gjøvik Gjøvik Norway; 3 Bundeswehr University Munich Neubiberg Germany; 4 Department of Osteopathy Faculty of Health and Social Sciences Hochschule Fresenius Munich Germany; 5 Tübingen University Children’s Hospital Tübingen Germany

**Keywords:** osteopathy, osteopath, osteopaths, osteopathic profession, health care system, Austria

## Abstract

**Background:**

There are no uniform regulations for the osteopathic profession in Europe. It is subject to country-specific regulations defining who shall be allowed to practice osteopathy and which qualification shall be required. In recent years, legal regulations have been established in several European countries for the profession of osteopathy; however, these are also still pending for Austria. Currently, physiotherapists and physicians with osteopathic training are practicing osteopathy in Austria.

**Objective:**

This study aims to examine the characteristics, challenges, and opportunities of osteopaths in Austria.

**Methods:**

Guideline-based interviews with osteopaths (N=10) were conducted. The different research questions were examined using a qualitative content analysis.

**Results:**

The study provided a differentiated insight into the professional situation of osteopaths in Austria. The most important result was that all interviewees unanimously supported a legal regulation of their profession. However, owing to their different professional self-image—on the one hand, individuals working on a structural basis, and, on the other hand, individuals working on a cranial or biodynamic basis—they were able to imagine a uniform professional regulation only to a limited extent. Additional topics for the interviewed osteopaths in Austria were the quality assurance of training and the urgent need for scientific research. Furthermore, the study also dealt with the influence of the COVID-19 pandemic on daily practice and on education and training in osteopathy.

**Conclusions:**

This study is a pioneering study with regard to systematic basic research on osteopathy in Austria. The obtained results and the newly acquired research questions not only have the potential to serve as a basis for further studies but also provide insight into the working and professional situation of osteopaths in Austria for universities, schools, professional associations, politics, and—last but not least—all interested parties.

**International Registered Report Identifier (IRRID):**

RR2-10.2196/15399

## Introduction

Osteopathy is a manual treatment method, the principles of which are based on its own philosophy and the consideration and treatment of special structure-function relationships in the human body [1]. Since osteopathy was established and as long as it has been applied, both its methods and the professional competence of osteopaths have been the subject of controversy among medical and therapeutic specialists. The European Committee for Standardization has defined osteopathy as a holistic, patient-centered, manual treatment method based on the interactions between the structure and function of the body and the body’s self-healing ability [2].

There is no uniform European or international regulation regarding who is allowed to practice osteopathy and which qualifications are required. However, an increasing number of European countries are developing occupational laws for osteopaths. So far, 12 European countries including Cyprus, Denmark, Finland, France, Iceland, Liechtenstein, Luxembourg, Malta, Norway, Portugal, Switzerland, and the United Kingdom have adopted legal regulations regarding the practice of osteopaths [3].

In the German-speaking countries, there is no uniform picture of the profession. In contrast to Switzerland, a legal basis for the profession of osteopathy does not exist in Germany or Austria. In Austria, physicians and physiotherapists trained in osteopathy practice as osteopaths. Physicians are allowed to practice osteopathy without any restrictions, whereas physiotherapists are only allowed to practice osteopathy upon medical assignment [4]. According to the Austrian Society for Osteopathy (Österreichische Gesellschaft für Osteopathie; OEGO), approximately 2000 osteopaths practiced in Austria in 2022.

Studies about osteopathic identity are progressing internationally. However, the various legal regulations and intraprofessional conflicts make it difficult to perceive a collective identity [5]. Especially in countries where osteopathy is not regulated by law, the data about osteopathic practitioners are considered to be weak. However, quantitative studies that have surveyed the population of osteopaths with regard to work status, training, professional identity, or characteristics of clinical practice such as the typical patient profile and the use of diagnostic and treatment modalities exist already. In Austria, 2 surveys of osteopaths have been conducted in the past as part of final theses [6,7]. In 2022, the results of the Osteopathic Practitioners, Estimates, and Rates survey were also published for Austria, thus creating a solid data basis about osteopathic practitioners in Austria for the first time. The typical osteopath was defined as female, aged between 40 and 49 years, self-employed, worked before as a physiotherapist, trained in osteopathy part time, and successfully completed a master’s degree [8]. However, there is a lack of studies with qualitative designs to capture and examine the work of osteopaths in German-speaking countries in more detail.

The overall aim of this study was to make a substantial contribution to the largely unexplored profession of osteopathy in the German-speaking countries. Structured, basic research was necessary to implement this project. The first steps were taken in the framework of the study, “Characteristics, Opportunities, and Challenges of Osteopathy (COCO) in the Perceptions of Osteopaths in Germany, Austria, and Switzerland: Protocol for a Comprehensive Mixed Methods Study.” The study protocol was published in *JMIR Research Protocols* in 2019. The Characteristics, Opportunities, and Challenges of Osteopathy (COCO) project investigates how osteopaths in Germany, Austria, and Switzerland distinguish themselves from other medical professions and the characteristics of their work.

This study is a partial study of the COCO project, with a focus on the situation of osteopaths in Austria. Osteopaths practicing in Austria were asked about their professional profile and their professional practice. The following questions were of particular interest: (1) How do osteopaths from Austria describe osteopathy? (2) What are the challenges faced by osteopaths in Austria? and (3) What opportunities do the interviewees see for osteopathy in Austria?

## Methods

### Design

This qualitative study included the planning and implementation of guideline-based interviews with osteopaths practicing in Austria. Subsequently, a qualitative content analysis was performed according to Mayring [9]. A qualitative research design was selected to obtain questions relevant to the project that had not been considered before and views about the topic that had not yet been taken into consideration. The target of this qualitative partial study was the development of hypotheses. Accordingly, a relatively small sample of 8 to 10 participants could be used, because the results obtained shall be examined in subsequent studies with respect to their general validity using a quantitative study design [10].

To ensure the reporting quality regarding the research methodology of this qualitative study, COREQ (Consolidated Criteria for Reporting Qualitative Research) was used [11]. A checklist including the COREQ items taken into consideration has been attached to the paper (Multimedia Appendix 1). The registration identifier of the study is the International Registered Report Identifier: PRR2-10.2196/15399.

### Ethical Considerations

**Figure 1:**
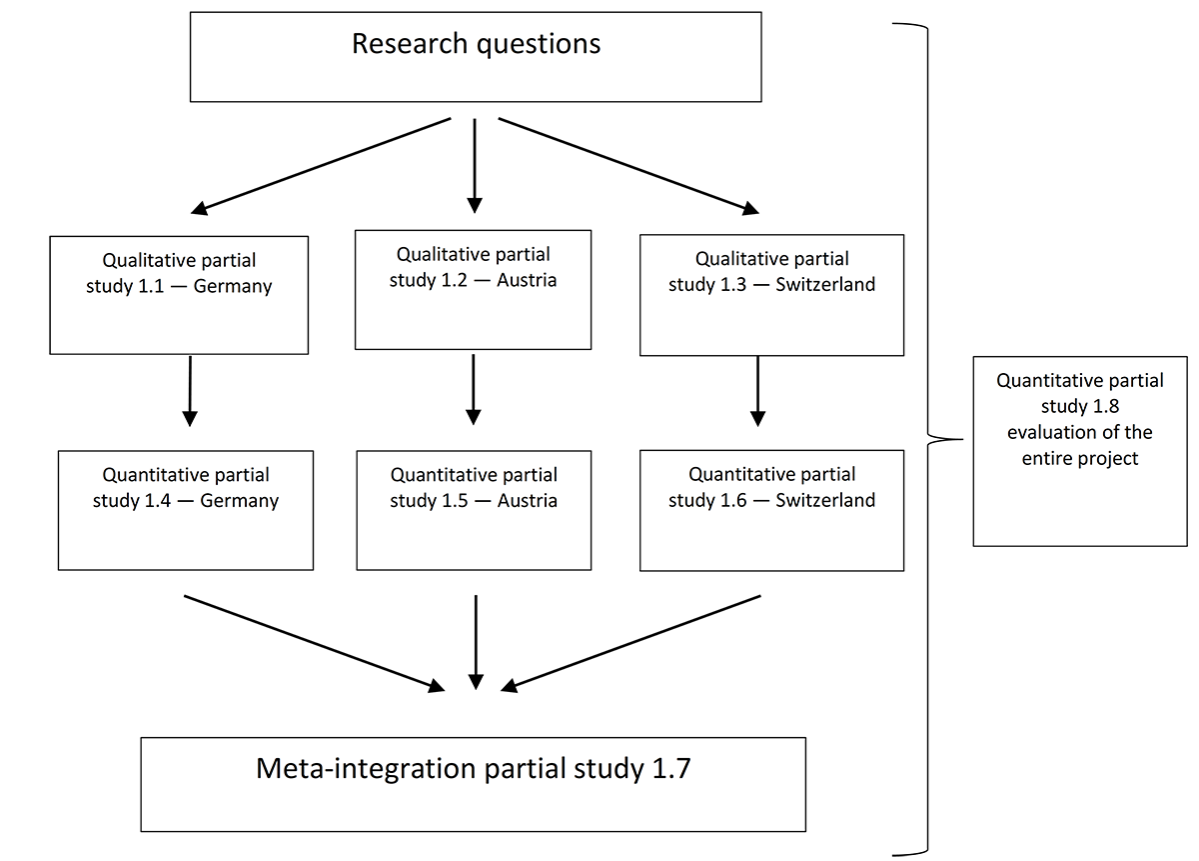
Characteristics, Opportunities, and Challenges of Osteopathy project flowchart.

This study (corresponding to partial study 1.2 in Figure 1), led by DM, has received ethics approval (S-287/2020) from the ethics committee of the University of Witten/Herdecke, Germany. Participants were not compensated for their participation.

### Setting and Sampling

Related to the research topic, osteopaths in Austria were questioned through guideline-based interviews. All the interviewed osteopaths (10/10, 100%) had completed at least 4 years of training as osteopaths and practiced as osteopaths in Austria. Instead of asking individual osteopaths to participate, OEGO was contacted with the study project itself, thus avoiding cold-calling. In this way, the criterion of comprehensive training in osteopathy was fulfilled, because otherwise, the participants could not be members of the professional association. This procedure ensured that the participants had provided evidence of their competence. To obtain the widest range of views, the sample was intended to show a high degree of diversity among the participants. Therefore, a further criterion for the whole group of participants was sex distribution according to the population of osteopathic practitioners in Austria. According to the OEGO’s membership register, two-thirds of practicing osteopaths in Austria are female and one-third are male. Moreover, care was taken to ensure that the residences and workplaces of the participants were subject to as wide a geographical distribution as possible, so that district-specific phenomena could be excluded. As osteopathy is not an independent profession in Austria, the participants should include the different occupational groups that practice osteopathy. Inclusion and exclusion criteria were subsequently formulated (Textbox 1).

OEGO forwarded the contact details of 11 osteopaths. Appointments for an interview were made with 8 (73%) of the 11 osteopaths. No appointment could be made with 1 osteopath during the study period. From 1 other osteopath, no response to the request was received. Another 1 osteopath did not want to participate in the study; 2 new osteopaths were suggested by the osteopaths themselves. A total of 10 interviews were thus conducted.

All the participants were contacted via email and received a letter containing information about the course of the study, declaration of consent to participate in the study, and data protection declaration. The entire participation in the research project and the answering of individual questions was on a voluntary basis; nonparticipation did not lead to any disadvantages for the participants. The participants always had the option to end the survey (eg, in the case of unexpected, stressful questions). Through the format of web-based survey, any increased risk of infection for the participants owing to the COVID-19 pandemic could be excluded. Explicit cancellation criteria for the project were not set.

**Inclusion and exclusion criteria.**

**Inclusion criteria**

- The individual has completed 4 years of osteopathic training.
- The individual is currently practicing osteopathy.
- The individual has provided consent to participate in the study.

**Exclusion criteria**

- The individual cannot be interviewed within the examination period.
- The individual does not have the technical equipment required for participation in a web-based survey.
- The individual demands compensation.

### Development of the Interview Guidelines and Data Collection

The guideline-based interview was chosen as a suitable research tool, because the aim of the data collection was to obtain concrete statements about the practice of osteopathy in Austria. In addition, the use of a guideline increased the comparability of the individual data sets [12]. Furthermore, this procedure avoided the possibility that essential aspects of the research question might be overlooked in the interviews [13]. The interview guideline was developed by JM on the basis of the research questions and 2 previous qualitative, partial studies of the COCO project [14,15]. The interview guideline was developed by JM on the basis of the research problem and 2 previous qualitative, partial studies of the COCO project [14,15].

The questions were formulated as open questions (Textbox 2) and arranged according to the groups of topics (Textbox 3). In addition, sociodemographic data about the participants were collected. Alternative questions were prepared to be able to react flexibly to the course of the interview and to respond to the potential needs of the participants. To maintain the flow of the conversation, additional questions were developed in advance. Before beginning the interviews, a test interview was conducted with a German osteopath to test the interview guideline in practice and to improve the interview technique. Finally, the questionnaire was discussed and adapted together with JP, an osteopath with experience in qualitative research.

**Example interview questions.**

- Where did you first hear about the osteopathic profession?
- How would you define osteopathy?
- In your opinion, what differentiates osteopathy from other professions?
- What does a typical osteopathic treatment look like for you?
- Should osteopath be its own profession?
- Are there any difficulties or problems that you face in your daily work life as an osteopath?
- Has the corona pandemic changed anything in your daily practice?

**Contents of the interview guideline.**

**Training and work in osteopathy**

- Educational background
- Motivation
- Training structure
- Work experience
- Acquisition structure
- Fields of activity

**Characteristics of osteopathy**

- Definition
- Properties
- Differentiation of professional profile
- Competences
- Features
- Limits

**Challenges of osteopathy**

- Health value
- Employment policy
- Obligations
- Restrictions
- Conflicts

**Chances and opportunities in osteopathy**

- Perspective
- Research
- Desires

### Data Collection

Data were collected from November 29, 2021, to January 26, 2022. In total, 10 interviews were conducted. The interviews were conducted using the Zoom software (Zoom Video Communications; audio and video were recorded). Apart from research economy and temporal and local flexibility, interviews were primarily conducted on the web to protect the participants from infection during the COVID-19 pandemic. No other individuals were present during the interviews. No interview was repeated. The interviews were recorded as a video file for transcription. The participants agreed in writing to the archiving of the files until the end of the publication activities or up to a maximum of 5 years after data collection. The files were protected against unauthorized access and stored and evaluated on local data carriers of a password-protected computer. Only encrypted files were transferred among the study colleagues. Upon completion of the study project, the recordings of the interviews shall be deleted irrevocably. All the interviews were conducted by JM in German. The interviewer is male, holds a master of science degree, and has already published in 2019 within the COCO project. At the time of the survey, he worked independently as a physiotherapist in his own practice in Germany and was a doctoral student at the University of Witten/Herdecke. For this research project, JM was trained within a 3-part seminar at the Freie Universität Berlin regarding the collection and evaluation of qualitative data and the conduction of interviews. There was no previous personal relationship with any of the interviewees. The recordings were transcribed by JM. Transcription was performed according to pre-established rules, which were consistently observed, as there are no generally accepted transcription rules [16]. The rules were based on the transcription rules of Kuckartz and Rädiker [17] for computer-assisted evaluation. The participants were sent the transcript to gather their comments, if any, and to receive their final approval.

### Data Analysis

On the basis of the results of the interview studies already conducted within the COCO project, deductive (ie, indirectly theory-driven) categories were formed first. It is indirect because the categories are descriptive and their definition is not the basis of a theory-driven description. As a first step, classical deductive codes were derived from the interview guideline (deductive category application). The transcripts were analyzed in the original language by means of content structuring. With the help of the MAXQDA 2022 software (VERBI Software) [18], quotations that were relevant for the abovementioned research questions were categorized. Owing to an extensive interest in further knowledge, the preselected segments were expanded or specified by means of inductive categorization to deduce further important aspects (deductive-inductive categorization) [17]. The quotations were summarized in categories, which were subsequently grouped into high-level categories. The main strategies of data analysis are described in Textbox 4.

**Data analysis strategies.**

**Coding**

- After developing an initial category system together (first, deductive; second, inductive), 2 complete material iterations were performed by 2 separate evaluators

**Discussion**

- Comparison and discussion of the results, with the main focus on the integration of different perspectives and the elimination of ambiguities

**Quality control**

- To check the quality of the category system (intercoder reliability) with its coding rules by means of the Cohen κ coefficient

**Final iteration**

- After the quality control step, a final, complete material iteration was performed by an evaluator using the final category system

### Shared Coding

A first interview was coded by 2 evaluators together (JM and UW), and the category system was inductively expanded. An initial category system was developed, and coding rules were determined on the basis of 2 partial studies (eg, Figure 1—partial studies 1.1 and 1.3) [14,15] and the interview questions and guidelines developed specifically for this partial study. The result was an initial, deductive category system, and the first coding rules were defined. The deductively determined categories were based on the research questions and increased based on inductive subcategories during the evaluation. Then, the first iteration was run with the entire material, followed by further inductive categorization (UW). A second evaluator (JM) ran the second iteration of the entire material based on the category system resulting from the first material iteration. The results were then compared and discussed, with the main focus being on the integration of different perspectives and the elimination of ambiguities. The code definitions and anchor examples were also revised in this step. Finally, the category system was standardized. This shared coding from the beginning of the study was intended to increase the intersubjectivity of the statement.

### Quality Control

Subsequently, of the 10 interviews, 3 (30%) were selected via lot procedure as a subsample to check the quality of the category system with its coding rules by means of the Cohen κ coefficient. The determination of the Cohen κ coefficient is a method for checking the intercoder reliability [19]. UW received the 3 allotted coded interviews from JM. The segment boundaries of the encodings were retained during this iteration. In this way, the evaluator was able to recognize which text parts were encoded and arrange these according to the categories. Without random adjustment, a match of 62.1% between the 2 encodings of this subsample was reached. The randomly adjusted coefficient was 0.61. Pursuant to Altman [20], accordance is considered as “good” in the case of a κ coefficient of 61-80. After this quality control, as there was good accordance; a final complete material iteration was performed by an evaluator (JM) using the final category system.

## Results

### Overview

The result of this study was a system of categories in which the statements of the participants were classified and subcategorized according to the research questions regarding the groups of topics, such as characteristics, challenges, and opportunities of the osteopaths practicing in Austria. Sociodemographic data and more general statements about the practice of osteopathy were also classified in the main category, “training and work in osteopathy.” Out of current concern, the osteopaths were also asked about the influence of the COVID-19 pandemic on their work. On the basis of the interesting statements, we decided to dedicate a special category to this topic.

The final category system consists of 71 categories with a total of 783 encoded text passages. A definition was created for each individual category, and a quotation was recorded as an anchor example.

Only a part of these results could therefore be described in this paper. The printed quotations have been translated into English and serve for illustration purposes only. The question whether individual statements of the participants (O1 to O10) represent the entire population of osteopaths practicing in Austria shall be subject to further investigation.

### Training and Work in Osteopathy

A total of 10 osteopaths practicing in Austria were interviewed—6 (60%) women and 4 (40%) men. Of the 10 osteopaths, 3 (30%) were physicians and 7 (70%) were physiotherapists. Of the 10 interviewees, 9 (90%) worked independently in their own practices and 1 (10%) had an employment relationship at the time of the interview. Only 10% (1/10) had other employees. The longest interview lasted 61 minutes, and the shortest interview lasted 29 minutes. The average duration of the interviews was 46 (SD 9.2) minutes. The participants’ characteristics are described in Table 1.

**Table 1:** Participants’ characteristics (N=10).

Characteristics		Participants, n (%)
**Sex**		
	Male	4 (40)
	Female	6 (60)
**Profession**		
	Physician	3 (30)
	Physiotherapist	7 (70)
**Degree**		
	Diploma in osteopathy	3 (30)
	Master of science	6 (60)
	Nondegree	1 (10)
**Training facility**		
	Vienna School of Osteopathy, Vienna, Austria	8 (80)
	European College of Osteopathy, Munich, Germany	1 (10)
	The International Academy of Osteopathy, Darmstadt, Germany	1 (10)
**Clinical experience (y)**		
	1-5	2 (20)
	6-15	5 (50)
	>15	3 (30)

### Characteristics of Osteopathy

On the basis of the descriptions provided by the participants regarding the properties of osteopathy, the following categories were developed: “definition of osteopathy,” “patient profile,” “anchor personalities and literature,” and “limits of the treatment technique.”

#### Definition of Osteopathy

When asked about the definition of osteopathy, several participants had difficulties in explaining the concept:

Yes, it’s really a difficult question.

O1; item 35

The explanation of the term was mostly based on the manual work, origin of the word, differentiation or overlapping with other professional groups, or citation of definitions of third parties. Often, reference was made to the philosophy of osteopathy, holism of the treatment method, and activation of self-regulating forces. There was no uniform definition of osteopathy among the answers of the interviewees.

#### Patient Profile

Most of the interviewed osteopaths treat patients of all ages:

Oh, everything actually, there are patients of all ages. From...three-month-old babies to over 90-year-old men, women, so I couldn’t paint a typical picture.

O2; item 54

Common indications for child treatments mentioned by the interviewed osteopaths are sleep disorders, torticollis, scoliosis, asthma, abdominal colic, and plagiocephaly. The treatment of adults was mainly based on the diagnosis or leading symptoms from the orthopedic area: back and neck pain and joint pain. Neurological diseases such as Parkinson disease were also mentioned. Moreover, patients were regularly treated for headache, migraine, tinnitus, chronic pain, craniomandibular dysfunction, abdominal pain, and hormonal imbalances or if wishing to become pregnant. However, internal diseases such as sinusitis, bronchitis, and cystitis were also treated by the physician O2 on the basis of osteopathic methods.

The indication of the treatment was usually given by the patients’ treating physician:

Patients are often assigned by the doctors, meaning that the doctor writes a prescription with a recommendation to contact a certain therapist.

O8; item 51

A participant working as a general practitioner in addition to his osteopathic activity also acquired patients during his regular consultation as a physician:

And actually, many of those who go to the general practitioner’s clinic in the village, they come to me, too.

O2; item 54

It appears that, in general, a broad medical field was covered by osteopathy. The selection of the appropriate therapist seemed to depend on personal recommendations of others, on the therapeutical possibilities in the patients’ vicinity, and on the training or specializations of the osteopaths:

Because the patients who come to me come by word of mouth, yes.

O5; item 47

Everything else is, I think, very average, that is, all the people who come to me do so because I am in their vicinity.

O3; item 38

Patients who travel a long way mainly come because of endocrinological, metabolic problems..., gynaecological problems..., that is to say, where...the focus of my...training has mainly been during recent years.

O3; item 38

#### Anchor Personalities and Literature

It was noticeable that many participants referred to other individuals when answering questions about osteopathy in theory and practice. These “anchor personalities” seem to have a great influence on the self-image of osteopaths in Austria and have therefore been included in a separate category. Both historical personalities from the history of osteopathy and currently active osteopaths were repeatedly mentioned:

But there will always be people who really care about this innermost quality of osteopathy.... And just as osteopathy has developed from Still to Sutherland, Becker, Viola Frymann...and all their names..., or Mitchell and Jim Jealous now..., so it will continue to develop.

O5; item 84

With regard to the self-study of osteopaths, primarily only the German-language journals of osteopathy were mentioned as reading material. Of the 10 participants, only 1 (10%) indicated that they regularly read an English journal:

Yes, I regularly read the two journals, DO and Osteopathische Medizin.

O5; item 88

#### Limits of Osteopathy

There was agreement regarding the limits of osteopathy. The primary treatment of structural injuries or the care of patients with cancer without medical supervision were clearly mentioned by the interviewees as limits of osteopathic activity. However, patients were given osteopathic treatment nevertheless:

Yes, of course I see the limits in the pathologies that are there. If there is actually...an osteoarthritis that is simply there and will not vanish, osteopathy shall certainly have its limits; one can perhaps relieve the pain, but the osteoarthritis cannot be cured by osteopathy, now can it. I also see limits in some diseases.

O7; item 56

O9 differentiated upon request that it is not the diagnosis that is decisive for the objective of treatment when it comes to whether osteopathic treatment is indicated or contraindicated:

Well, that depends on the objective.... It all depends totally on the objective. If I say that I want to treat coxarthrosis curatively, I think that we shall soon reach our limits with osteopathy; if we do a control X-ray after six months, we shall see that it is still coxarthrosis.... But if I say that I want to improve the quality of life, I would treat them nevertheless. The question is what the objective is.

O9; item 41

O4 brought another aspect to mind:

We must not exceed the limits...of our own competence. That is very important. Unfortunately, many colleagues do this by suddenly giving dietary recommendations, by suddenly recommending medicines...or by talking patients out of taking medicines. Especially now when it comes to vaccination.

O4; item 75

### Challenges of Osteopathy

The challenges mentioned for the field of osteopathy can be classified mainly into the categories, “identity problem,” “disagreements within the osteopath community,” “research,” “training quality management,” and “conflicts and difficulties.”

#### Identity Problem

One of the great challenges of osteopathy is its unclear definition and lack of differentiation from other professional groups. As O6 clearly pointed out, osteopathy has an identity problem:

I would say that the identity problem is the most important issue...The definition is the most difficult question and no one can answer that. And if we can’t answer that and don’t deal with it, how can we argue what we are if we ourselves don’t know exactly what we are.

O6; item 49

#### Disagreements Within the Osteopathic Community

The identity problem or the problem of missing a uniform professional self-image might be based on disagreements within the osteopathic community described by several participants. Overall, 2 groups can be identified among practicing osteopaths: on the one hand, the structurally working osteopaths and, on the other hand, the cranially or biodynamically working osteopaths:

Yes, there is really a gap between biodynamic osteopathy and structural osteopathy.

O7; item 104

This conflict might be decisive not only in terms of a common definition but also with respect to a possible recognition of osteopathy in terms of professional policy. O9, who was involved in professional policy, feared that these disputes might even prevent recognition:

The problem concerning regulation is - and that’s simply the case now and that’s also the elephant in the room about which no one is talking -...the problem with regulation has always been cranio.... You cannot say it openly, but it was always the problem of craniosacral therapy, no matter who I talked to.

O9; item 93

Those participants who worked biodynamically were more critical toward regulation:

If one tries now to take this out of this mental...source,... I see the risk that it is practically shifted into evidence-based, as important as that is, well, but only into evidence-based, visible and perceptible dimensions, then osteopathy shall lose its soul from my point of view.... And that’s actually the greatest threat to osteopathy for me.

O5; item 64

#### Research

In this context, O1 pointed out that evidence-based research can only substantiate a certain part of osteopathy scientifically, whereas other aspects might be lost:

A good scientific basis in order to argue how...many benefits osteopathy has...- in the end,... academisation probably cannot be avoided and will certainly be necessary. Even if all these developments are not entirely without risks. That is, the risk of losing sight of the holistic aspects of osteopathy.

O1; item 81

#### Training Quality Management

Many osteopaths considered existing weaknesses in the training courses and their structures as a further obstacle. According to O4, a central aspect is the inadequate teaching of the skills for scientific work at osteopathy institutes and, thus, the lack of evidence-based research in osteopathy:

[Oh my] the training...We should learn from the beginning, not only during the last year when we have to write a Master Thesis, we should learn from the beginning what it means to work in an evidence-based way, to do research...It works in physiotherapy and is continuously getting better there, but in osteopathy...At the beginning, we never learn to deal with available studies, it is a matter of training. From the beginning, not only during the fifth year shortly before the Master Thesis, we should have the first lessons in statistics.

O4; item 113

#### Conflicts and Difficulties

The lack of clarity regarding the profession is evident in the differentiation with respect to other professional groups. With regard to the settlement for osteopathic treatment with health insurance providers, several participants also reported potential for conflicts. Osteopathic treatment is often provided on the grounds of a physiotherapeutic prescription by the physician. The reasons are the economic pressure on the practices or the social situation of the patients:

Many colleagues work as physiotherapists, they also charge for osteopathy as physiotherapy, and yes, they are refunded in this way. And as a result,...osteopathy is also a little...less in the focus than it should be. I’ve been working for 20 years now, I’m only writing osteopathic invoices.... But...of course...I understand the problem. If somebody has fewer patients...and...has to charge for...physiotherapy, I absolutely understand the situation.... But...these problems are of course...long-burning issues.

O5; item 78

No, [the bill] of course says physiotherapy and remedial massage, because otherwise the patient doesn’t get his/her money from the insurance company. For the insurance company, well, this is ok or it is tolerated. I have already received the feedback from many patients that they told the company that they went to an osteopath, and the health insurance said that of course that can’t be billed, [but] we shall write physiotherapy and remedial massage and then that’s it.

O8; item 55

When asked about the challenges for osteopaths in general, the physicians working as osteopaths did not report any difficulties related to their practice. A physician and osteopath, in contrast, was aware of the potential for conflict:

Yes, of course I know that. I have a bonus, because I’m simply a doctor. And of course, osteopaths that aren’t doctors have greater difficulties and are often rejected,...well,...because they are no medical doctors in a manner of speaking and...there are obviously difficulties.

O1; item 101

Another participant stated it even more clearly:

No, I’m a doctor, I have...no restrictions.

O9; item 85

### Opportunities in Osteopathy

Regarding the questions about the opportunities and chances in osteopathy, most of the statements could be classified into the categories “professional profile” and “position in the health care system.” A central opportunity in osteopathy is the installation of an independent profession. Almost all the osteopaths explicitly formulated the desire for their own professional profile. However, there was disagreement about the questions regarding where and how this profession should be integrated into the health care system or which competences it should include:

In the midst of the other health professions..., well, I don’t see us as special consultants, as it is now in America, for example. But I see us as a health profession next to physiotherapists, occupational therapists...

O4; item 81

In this context, many possible applications were mentioned for the field of osteopathy. An osteopath saw a great opportunity in the prevention of diseases:

Concerning also prevention..., I believe that osteopathy has an enormous potential for people’s health by simply doing something really good and also really preventing things,...follow-up problems or operations or God knows what...I see a huge opportunity there.

O10; item 107

O2 also attributed the potential for cost reduction to preventive osteopathy. From his point of view, examinations and medical consultations might be reduced:

I see a huge and very central importance of osteopathy in primary care...and I am convinced...from my daily experience that an incredible number...of diagnostic measures or specialist care...might be avoided if people were primarily also treated by osteopaths.

O2; item 68

Whether osteopathy actually contributes to disease prevention and can thus also lead to cost reduction or relief for the health care system is to be investigated using clinical study designs on the effectiveness of the treatment method itself. The position of osteopathy in the health care system and its differentiation from other professional groups have also not been uniformly described by practitioners in other countries.

### The COVID-19 Pandemic

For current reasons, the participants were questioned about the effects of the COVID-19 pandemic, existing since March 2020, on their professional activities. Similar to a magnifying glass, crises very often reveal the weaknesses and failures of structures and concepts; however, they can also show their viability and strengths. Most respondents described the time of the COVID-19 pandemic with the lockdowns and the associated measures as a turning point in their practice. However, none of the participants described economic losses or existential fear:

Well, the time during Covid-19 wasn’t easy at all.

O5; item 75

At the time of the survey, everybody had to wear a face mask, patients and osteopaths alike. The interviewees not only described the difficult communication with the patients because of the mask but also mentioned limitations during examination and treatment. Certain treatments, for example, techniques relating to the mandibular joint, could not be performed for patients wearing a face mask:

I believe that a lot of communication is lost through the mask, because you don’t see the whole face of the patient. Of course, you have communication through the eyes, but there is still a barrier, a lot is lost...It already starts with and continues during inspection: you only see half of the face and in the case of jaw problems, I have to take down the mask first.

O8; item 107

Almost all the interviewees described a change in the clientele of their patients. Stress, sleep disorders, headaches, and dysfunctions of the mandibular joint were increasingly mentioned:

Psychosocial stress is increasing immensely...This in turn results...in sleep disorders...Mandibular joint problems due to stress, but also - and this is my own observation - because you constantly want to push around this mask if you have to wear it all day...I think that this has a huge influence...[O6; item 79]

Teaching also seemed to be affected by the protection measures for pandemic control. An osteopath reported that teaching on inpatients at the hospital ceased. The question of whether the osteopathic treatment of inpatients in institutions was disturbed by a lack of external osteopaths remained unanswered:

Prior to the lockdown, our osteopathic child centre also paid visits to the neonatal ward...where we treated premature babies. Unfortunately, this is not possible at the moment.

O1; item 47

Owing to the cancellation of congresses and courses or their transfer to the digital world, interviewees experienced a gap in their personal training plans:

Of course, I...repeatedly attended courses. However, I scarcely did so in the last two years, actually...[O5; item 88]

This can only be a small insight into the impact of the pandemic in the field of osteopathy. The effects of the COVID-19 pandemic on osteopathic care should be investigated systematically in the next few years.

## Discussion

### Principal Findings

This study identified numerous aspects, possibilities, and opportunities in osteopathy in Austria from the point of view of the osteopaths practicing in Austria.

In our survey, the typical osteopath presents as female and has previously worked as a physiotherapist, as previous studies have found [8]. This is consistent with other surveys from Europe regarding osteopathy. Moreover, in accordance with a study from Italy, the typical osteopath practicing in Austria works independently in their own practice and without employees [21].

The osteopaths interviewed usually found it difficult to define osteopathy. The respondents were not able to provide a uniform definition of osteopathy. Many respondents even expressed difficulties in precisely describing their profession. Nevertheless, recurring patterns can be recognized in the explanations given by the respondents.

The participants attempted to define osteopathy by drawing a distinction or differentiation from other professions and using third-party definitions. Furthermore, the philosophy of osteopathy, various osteopathic concepts or models of thought, the holistic nature of the treatment method, and activation of the patient’s self-healing powers are often referred to. A possible reason for the heterogeneous attempts at explanation may lie in the difference in training and previous education. A recent study showed that only 17% of osteopaths surveyed in Austria identified themselves “exclusively” as osteopaths [8]. Therefore, there is a suspicion that, as our study also showed, the basic profession and nonregulation have a major influence on self-image. We observed a fundamental distinction between therapeutic and medical osteopaths.

From our point of view, the clear statements regarding the disagreements within the osteopath community were surprising. The conflicts do not remain in the specialist circles of osteopathy, but they even extend to the level of professional policy. The question is whether this is a country-specific observation for Austria. In their study in Australia in 2018, for example, Blaich et al [22] found disagreement about the specialization of osteopaths; however, it did not result in the splitting of osteopaths into 2 separate groups. The belief patterns and paradigms of individual treatment techniques that influence professional identity are not new in osteopathy [23]. The fact that, according to the osteopaths interviewed, these intraprofessional conflicts exist even on the political level or are the reason for nonregulation is remarkable. An increasing number of European countries regulate the professional practice of osteopathy. Therefore, it remains to be investigated whether this dispute itself has an influence on the nonregulation of osteopathy in Austria. However, conflicts and different opinions within a professional group are not inherent in osteopathy; these also exist in other medical professions such as chiropractic [24,25].

The general development of a profession is not only subject to cultural, historical, and social influences but also to the question of gender [26]. In this context, this study indicates a large influence of anchor personalities on the self-image of the interviewed osteopaths. It is remarkable that the anchor personalities mentioned are almost exclusively men. The historical context is worth noticing here, because Andrew Taylor Still, the founder of osteopathy, explicitly promoted equality between men and women already in the 19th century, in contrast to many other universities or teaching institutes during that time. He expressly included women in his courses [27]. In this context, it should be noted that in other health professions, although the practitioners are predominantly women, the leadership positions are often mainly occupied by men [28]. It is therefore not surprising that most users and practitioners of alternative medicine are women if their health needs are not being met by scientific medicine [29]. This becomes problematic when these professions are or become patriarchally dominated to match scientific standards [30].

To answer the questions about the origin of these conflicts and to deal with these in the future, we believe that a systematic and country-specific scientific analysis will be required. Conflicts in the health care system not only have the potential to weaken a profession but can also have a stimulating influence if understood as an opportunity [31].

Most of the osteopaths surveyed were in favor of a legal regulation of the profession. Under certain circumstances, osteopathically trained physiotherapists could benefit more from this, as they currently still need a physician’s order to be able to practice with legal certainty.

However, nonregulation also has also some advantages—no applications for licenses, no obligation for regular further training, and unregulated pricing for treatment. With integration into the health care system, some participants fear deterioration owing to possible low or lower payment by health insurance companies.

Training quality management and studies in the subarea of osteopathy were also mentioned as challenges in this context. In Italy, Sweden, and Australia, the transfer of scientific results to the practical work of osteopaths has already been systematically investigated in a country-specific manner [32-34]. The openness to evidence-based practice (EBP) appears to exist among practicing osteopaths on a transnational basis, but the skills in dealing with the former vary from country to country. A study of EBP from Spain characterized the skills of the participants to deal with EBP as being rather low. This might be related to the lack of legal regulations and the inadequate transfer of knowledge in the training institutions [35]. The situation regarding osteopathy is similar in Austria. Additional country-specific studies are required to identify conclusions and connections.

The different situation in everyday practice owing to the COVID-19 pandemic and the respective infection protection measures also had an influence on the daily work of osteopaths. Several interviewees realized an evident change in the patients’ profile. Although economic damage or fear for their professional existence were not explicitly described, most osteopaths working independently were themselves responsible for the implementation of the legal measures in their practices. The impact of the pandemic on the daily work in practice seems to have been less considerable than the impact on the field of training in osteopathy. As a large part of practical teaching occurs with patients under supervision, it is difficult to implement in a web-based format. The impact of the pandemic on clinical research at universities or universities of applied sciences remains to be examined.

In the case of further investigations in this area, we recommend a specific distinction of the participants between physicians and physiotherapists practicing as osteopaths. As there is no uniform training or legal regulation of osteopathy in Austria, only physicians and physiotherapists trained in osteopathy exclusively practice osteopathy. The results of this study suggest that there are evident differences between these 2 professional groups regarding, for example, patient acquisition, conflict management, and cooperation with other professional groups.

The extent to which the individual statements made by the interviewees represent the entirety of osteopaths practicing in Austria will be further investigated. The protocol of the COCO project describes the further procedures. The results of the qualitative partial studies (studies 1.1, 1.2, and 1.3 in Figure 1) will be combined in a following study to verify the results of the qualitative partial studies in relation to the population [36]. We will develop a standardized questionnaire as a measuring instrument.

An important feature of this study is the methodology, including 2 evaluators who completed the entire evaluation process. Through this approach, intersubjectivity increased and new, inductively formed categories were created. During this phase, many aspects of the research problems could be identified and categorized. The intercoder reliability was tested and found to be viable within this study. With another material iteration, the category system can be further refined, and the intercoder reliability can be further increased by optimized code definitions.

### Limitations

First, it should be noted that the results of this study do not necessarily allow conclusions to be drawn about the entirety of osteopaths in Austria, as this is not an evaluation of representative surveys with large numbers of participants. Nevertheless, certain tendencies seem to emerge when statements by osteopaths appear to be congruent, that is, confirm each other or complement each other in a meaningful way. The sample represents the entirety of osteopaths in Austria well. Most respondents were women and physiotherapists [8]. Nevertheless, bias cannot be dismissed with such a specific sample. However, they give an idea about how osteopaths in Austria think, and the results obtained can serve as a hypothesis for large quantitative studies to test.

### Conclusions

It is difficult to characterize the community of osteopaths in Austria conclusively. On the one hand, there is a great deal of agreement about the urgency regarding regulatory legislation for their profession, a necessary revision of training structures, and the specific promotion of scientific studies of osteopathy. However, when it comes to the concrete practice of osteopathy, deep trenches and even strong disputes have occurred among osteopaths.

The following question remains to be answered: what is “correct” or “true” osteopathy? If we consider that osteopathy has derived from various sources; that its founder did not give a final answer to the question about what he understood by osteopathy; and that each discipline is constantly developing, solely through the different osteopaths practicing, it appears that this question cannot be answered completely.

Apart from this issue, there is another and equally sensitive question, that is, whether and how the different parties can or even must be brought together for the regulation of their profession, which is desired by most of them. The different professional origins of osteopaths should also be considered. With regard to binding legal regulations, which would not least strengthen the professional image, mutual understanding seems to be imperative. Perhaps such an understanding might also lead to greater political weight for osteopathy, which it urgently needs, not only in terms of legal regulations but also to be able to promote important research projects.

The question arises as to whether the conflicts within osteopathy, in particular, with their possible professional-political consequences and the immense influence of the basic profession in the practice of osteopathy, are a country-specific phenomenon for Austria. However, there is a lack of studies in German-speaking countries with comparable qualitative designs to assess the work of osteopaths in more detail. We are therefore planning a meta-synthesis of qualitative studies with the aim of generating new theoretical insights from the accumulation of study results. Both the studies from the COCO project itself and other relevant literature can be used for the meta-synthesis.

To the best of our knowledge, the COCO project is the largest mixed methods study project on the osteopathic profession in German-speaking countries. The category system with its reliability check can be used as a basis for a repetition of the study. Such a research project would also be interesting if the profession was regulated formally and substantially in the near future. The results presented in this paper are not only intended to serve as a basis for further studies but also to provide universities, schools, professional associations, and politicians with an insight into the situation of osteopaths in Austria.

## Appendix

Multimedia Appendix 1 COREQ (Consolidated Criteria for Reporting Qualitative Research) checklist.

## Abbreviations

COCO: Characteristics, Opportunities, and Challenges of Osteopathy
COREQ: Consolidated Criteria for Reporting Qualitative Research
EBP: evidence-based practice
OEGO: Austrian Society for Osteopathy (Österreichische Gesellschaft für Osteopathie)
